# Hydrothermal synthesis of hierarchical microstructure tungsten oxide/carbon nanocomposite for supercapacitor application

**DOI:** 10.1038/s41598-023-48958-w

**Published:** 2023-12-08

**Authors:** Shanna Marie M. Alonzo, John Bentley, Salil Desai, Bishnu Prasad Bastakoti

**Affiliations:** 1 Department of Chemistry, North Carolina A&T State University, 1601 E. Market St., Greensboro, NC 27411 USA; 2 Department of Industrial and System Engineering, North Carolina A&T State University, 1601 E. Market St., Greensboro, NC 27411 USA; 3 Center of Excellence in Product Design and Advanced Manufacturing, North Carolina A&T State University, 1601 E. Market St., Greensboro, NC 27411 USA

**Keywords:** Inorganic chemistry, Materials chemistry, Chemistry, Materials science, Materials for energy and catalysis, Nanoscale materials

## Abstract

A hierarchical nanocomposite of carbon microspheres decorated with tungsten oxide (WO_3_) nanocrystals resulted from the hydrothermal treatment of a precursor solution containing glucose and tungstic acid. The dehydration of glucose molecules formed oligosaccharides, which consequently carbonized, turning into carbon microspheres. The carbon microspheres then acted as a spherical nucleus onto which WO_3_ nanocrystals grew via heterogeneous nucleation. The reaction product showed a phase junction of orthorhombic and monoclinic WO_3,_ which transitioned to mix-phase of tetragonal and monoclinic WO_3_ after a subsequent heat treatment at 600 °C in an inert condition. The electrochemical tests showed that incorporating WO_3_ onto the carbon (WO_3_/C) resulted in a three-fold increase in the specific capacitance compared to WO_3_ alone and a high coulombic and energy efficiencies of 98.2% and 92.8%, respectively. The nanocomposite exhibited supercapacitance with both Faradaic and non-Faradaic charge storage mechanisms. Electrochemical impedance spectroscopy showed a lower charge transfer resistance for the composite at R_ct_ = 11.7Ω.

## Introduction

Supercapacitors are widely recognized to fall into two categories based on their energy storage principle: electric double-layer capacitors (EDLC) and pseudocapacitors. In EDLC, when voltage is applied, ions from the electrolyte are attracted to the surface of the electrode, forming a double layer of charges and resulting in purely physical energy storage^1^. Carbon materials are often employed for EDLCs because of their high surface area, suitable pore size, good electrical conductivity, chemical stability, and versatility^2–4^. These properties, particularly the high surface area and chemical stability, aid in effective ionic physisorption during electrochemical processes. However, their drawback lies in their low specific capacitance and energy density^5^. Conversely, pseudocapacitors achieve energy storage through reversible oxidation–reduction (Faradaic) reactions at/near the electrode surface. The additional contribution from these chemical redox reactions during charge/discharge leads to a higher specific capacitance^6–8^. Among the various redox-active materials, transition metal oxides have gained significant attention as electrode materials due to their multiple oxidation states, which allow for improved pseudocapacitance^5,6,9^. Tungsten oxide (WO_3_), a transition metal oxide with multiple crystal phases, exhibits favorable attributes as a pseudocapacitor^10–12^. Not only can it undergo reversible redox reactions between W^5+^ and W^6+^ ions, but the inherent voids in its crystal structure facilitate the smooth diffusion of ions from the electrolyte^13^. However, their limitations include relatively poor conductivity in bulk form and the tendency to aggregate during the charge/discharge process even in their nanostructure form, as is typical of transition metals^14^.

WO_3_ and carbon-based composites have been explored to address the abovementioned constraints in recent years. Combining WO_3_ and carbon materials creates a synergistic effect that complements each other's limitations, leading to better overall performance^15^. The hybrid presents a viable approach to enhance the electronic conductivity of WO_3_, improve the capacitance of carbon by incorporating redox reactions, lessen the aggregation of WO_3_ nanocrystals, and provide overall structural stability^9^. Most of these works involved nanotubes, nanowires, nanoplates, and nanosheets prepared in non-aqueous solutions or with expensive polymeric templates^16^. Nayak et al. utilized a solvothermal approach to synthesize a WO_3_ nanowire–graphene sheet composite^17^. Xiong et al.^18^ and Shi et al.^19^ prepared hierarchical ordered porous WO_3_–carbon using discarded biomass as a precursor, with the former using glue milling and carbonization-activation method and the latter via a solvothermal process. Di et al. also used a solvothermal technique to decorate carbon nanotubes with an array of WO_3_ nanosheets^20^.

This work synthesized carbon microspheres decorated with WO_3_ nanocrystals via a facile hydrothermal method using glucose as the carbon source. The simple procedure resulted in a hierarchical micro/nanostructure that could facilitate and enhance electrochemical reactions^21^. The crystal phase transformation of WO_3_ in the presence of glucose and its effect on the capacitive behavior of the WO_3_/C electrode was also investigated. Electrochemical tests revealed that the WO_3_/C nanocomposite provided more pathways for charge diffusion within its structure. These pathways appear to result from a cooperative interplay between the intricate nanocrystalline mixed phase WO_3_ and the porous carbon microsphere.

## Experimental section

### Materials

Analytical/reagent grade tungstic (VI) acid (H_2_WO_4_, Alfa Aesar), D( +)-glucose (Acros Organics), Nafion® D-521 (Alfa Aesar), ethanol (VWR Chemicals), and potassium hydroxide (Sigma-Aldrich) were used without further purification.

### Synthesis of the WO_3_/C composite and preparation of working electrodes

**Scheme 1:**
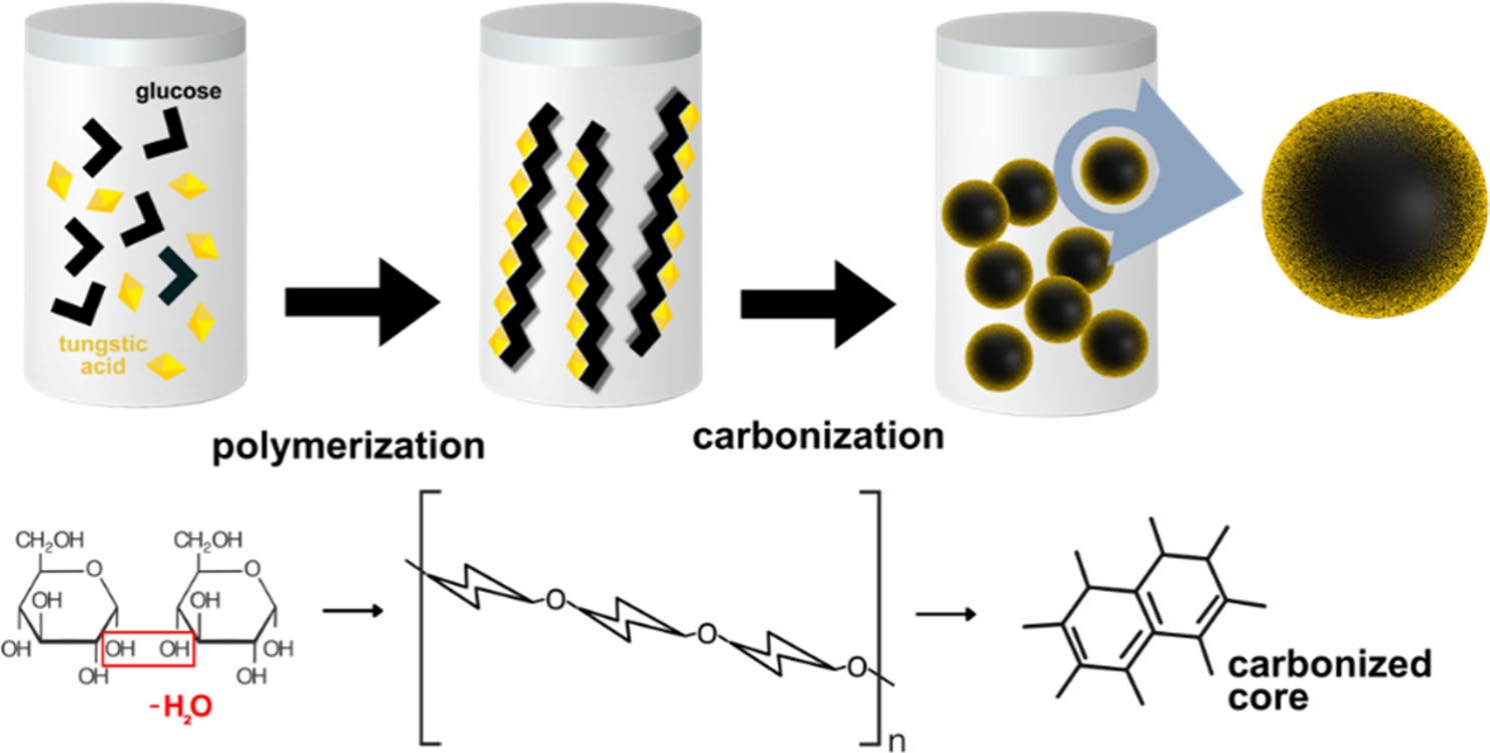
Graphical illustration of the WO_3_/C nanocomposite synthesis via a hydrothermal treatment strategy.

The precursor solution consisted of 1 g H_2_WO_4_ (dissolved in 5 mL ethanol), 1 g glucose, and 75 mL distilled water. The solution was transferred to a Teflon-lined stainless-steel autoclave, sealed, and heated at 180 °C for 20 h. After cooling, the hydrothermal reaction product was washed with water and ethanol before drying at 60 °C. Scheme 1 shows a graphical illustration of the hydrothermal synthesis. Carbon microspheres would form due to dehydration and oligosaccharide formation. Supersaturation would then lead to nucleation and subsequent growth of WO_3_ nanocrystals on these spheres via heterogeneous nucleation^14,22^.

The dried as-prepared sample was calcined at 600 °C for 3 hours at a ramping rate of 3°C/min in a nitrogen environment. A control sample without glucose was also prepared for comparison. 5 mg of the calcined powder sample was ground and dispersed in 500 µL ethanol to make the working electrodes. Then, 50 µL of the binder Nafion® D-521 was added. After mixing and sonicating, 220 µL of the slurry was drop-cast on copper substrates with a working area of 1.0 cm^2^ and dried at 60 °C for 12 h.

### Characterization

The crystal structure of the nanocomposites was studied by X-ray diffraction (XRD) using a Rigaku MiniFlex 600 diffractometer equipped with a Cu K_α_ radiation source and a scintillation counter detector. Powder XRD patterns were recorded from 10° to 60° (0.02° step, 2°/min speed) at 40 kV and 15 mA. Fourier transform infrared (FTIR) spectra were recorded from 400–4000 cm^−1^ using a Shimadzu IRTracer-100 spectrophotometer with a DLATGS detector. The Raman and X-ray photoelectron spectra were collected using a Horiba XploRA Raman confocal microscope and an ESCALAB™ XI + X-ray photoelectron spectrometer, respectively. The morphology and elemental mapping of the samples were investigated using a JEOL JSM-IT800 Schottky field emission scanning electron microscope (FESEM). For surface area and porosity analysis, N_2_ adsorption–desorption isotherms were measured at 77 K using Quantachrome NovaWin. The electrochemical measurements were performed using a CH Instruments workstation with a three-electrode configuration. Copper foil, Ag/AgCl, platinum wire, and the synthesized materials were used as the current collector, reference electrode, counter electrode, and working electrodes, respectively. The electrolyte used was 0.1 M potassium hydroxide. The electrochemical tests included cyclic voltammetry (CV), galvanostatic charge–discharge (GCD), and impedance spectroscopy. The specific capacitance was calculated using Eq. (1), coulombic efficiency using Eq. (2), and energy efficiency using Eq. (3),

1 $$C_{s} = \frac{{\mathop \smallint \nolimits_{{V_{1} }}^{{V_{2} }} Idv}}{ 2sm\Delta V}$$

2 $$\eta _{c} = \frac{{t_{d} }}{{t_{c} }} \times 100$$

3 $$\eta _{E} = \frac{{E_{int/d} }}{{E_{int/c} }} \times 100$$

where *C*_*s*_ is the specific capacitance (F/g), $\mathop \smallint \limits_{{V_{1} }}^{{V_{2} }} Idv$ is the integral CV curve area (AV), *s* is the scan rate (V/s), *m* is the mass of the active material (g), *∆V* is the potential window, *η* is the coulombic efficiency (%), *t*_*d*_ is discharging time (s), *t*_*c*_ is charging time (s), *E*_*int/d*_ is the galvanostatic discharge energy, and *E*_*int/c*_ is the galvanostatic charge energy^21,23,24^.

## Results and discussion

**Figure 1:**
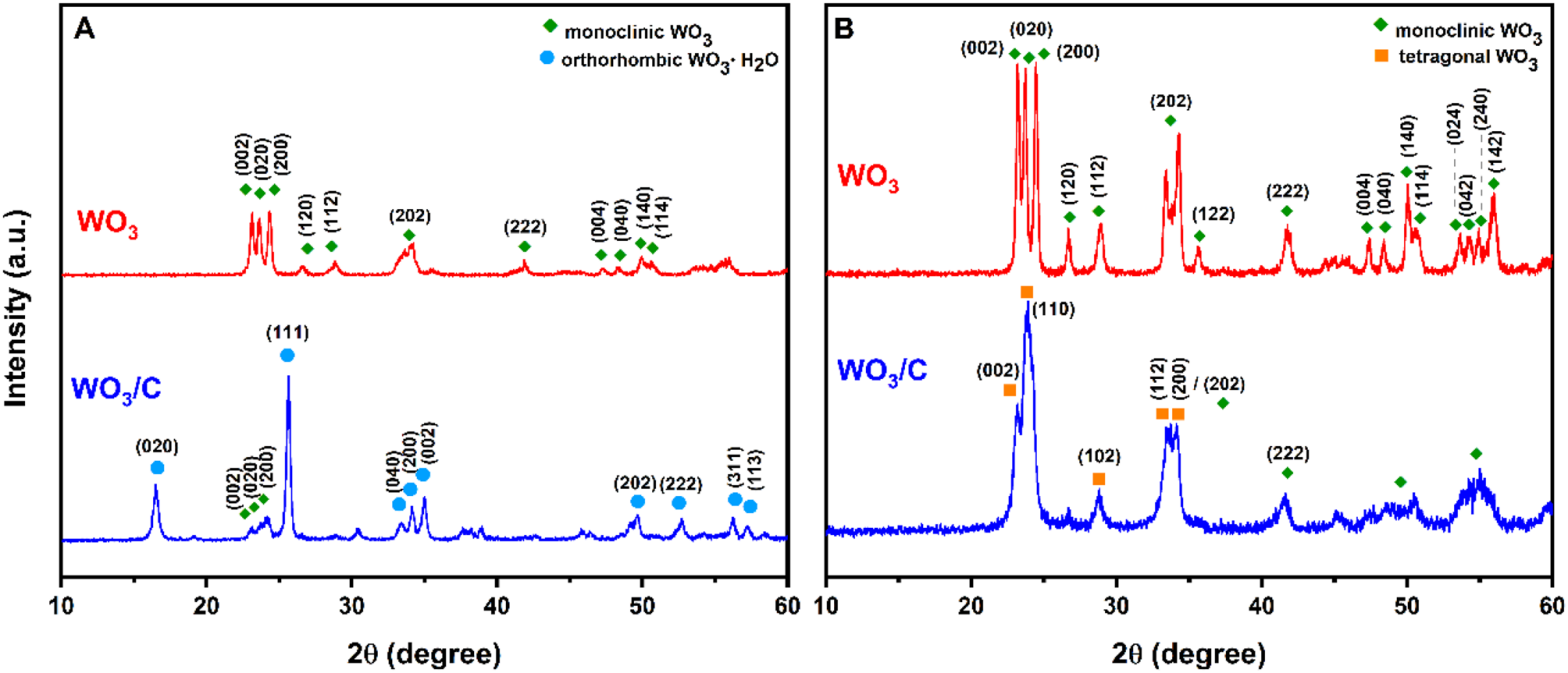
XRD patterns of WO_3_ and WO_3_/C (**A**) before calcination and (**B**) after calcination.

The samples’ XRD patterns showed sharp and intensive peaks, indicating a crystalline structure. In Fig. 1A, the XRD pattern of the uncalcined WO_3_/C composite (blue) is consistent with the orthorhombic crystal structure of WO_3_ (JCPDS No. 43-0679) and diffractograms reported in the literature^25–27^. The strong peaks at 16.5° and 25.6° are attributed to the (020) and (111) reflections of the orthorhombic crystal structure of tungsten oxide hydrate (WO_3_·H_2_O), respectively. These peaks were not observed in the control sample WO_3_ (Fig. 1A, red)_,_ which did not have glucose as a carbon precursor, suggesting that glucose aided in preserving the orthorhombic crystalline phase of WO_3_·H_2_O during the hydrothermal process. The hydroxyl group in glucose and the hydrogen in the WO_3_·H_2_O molecule formed a hydrogen bond, promoting the controlled growth of WO_3_·H_2_O crystallites^28^.

Moreover, the triplet peaks near 35.0° for the WO_3_/C nanocomposite (Fig. 1A, blue) could be indexed to the orthorhombic (040), (200), and (002) crystal planes. But in the uncalcined WO_3_ sample (Fig. 1A, red), these peaks appeared as a doublet at 34.1° and corresponded to the (202) plane, indicating a monoclinic structure^29–31^. The uncalcined nanocomposite WO_3_/C showed a coexistence of orthorhombic and monoclinic phases, with the former as the dominant phase, and the control WO_3_ exhibited a purely monoclinic phase. The sharp diffractive peaks at 23.1°, 23.7°, and 24.2° corresponded to the (002), (020), and (200) crystal planes, consistent with JCPDS No. 43–1035 for monoclinic WO_3_. After calcination, as shown in Fig. 1B (red), the control WO_3_ did not change its phase but exhibited increased crystallinity with peaks becoming sharper and more defined. For instance, the doublet peak at 34.1° diverged more clearly. The elevated temperature during calcination provided sufficient energy for adjacent tiny crystals to rearrange and coalesce into larger crystals^32,33^. This increase in crystallite size of WO_3_ from 16 Å to 23 Å after calcination appeared as narrower, more intensified XRD peaks. On the other hand, the WO_3_/C composite changed its phase after calcination (Fig. 1B, blue). The intense peaks at 16.5° and 25.6° characteristic of orthorhombic crystal, disappeared, indicating the removal of the hydrate water in WO_3_·H_2_O^25^. The XRD pattern after calcination showed a tetragonal/monoclinic phase junction. The main diffraction peaks at 23.0°, 23.9°, 28.7°, 33.5°, and 34.0° could be attributed to the (002), (110), (102), (112), and (200) planes of tetragonal WO_3_, aligning with COD No. 1521532^34,35^. However, this attribution may not be absolute, and a pseudo-phase consisting of orthorhombic and tetragonal phases would also be likely, as observed from previous work^13,34^. The less defined peaks from 45° to 60° resembled that of monoclinic WO_3_ and indicated a reduced intensity due to the amorphous carbon. The energy storage performance of WO_3_ depends on its crystal structure which influences the intercalation of ions in an electrochemical environment^5,9^. Orthorhombic and tetragonal WO_3_ generally tend to have more cavities or open spaces within its crystal structure than monoclinic WO_3_. The more open structure and wider tunnels in the former allow fast, reversible intercalation of ions^5,9,36^.

**Figure 2:**
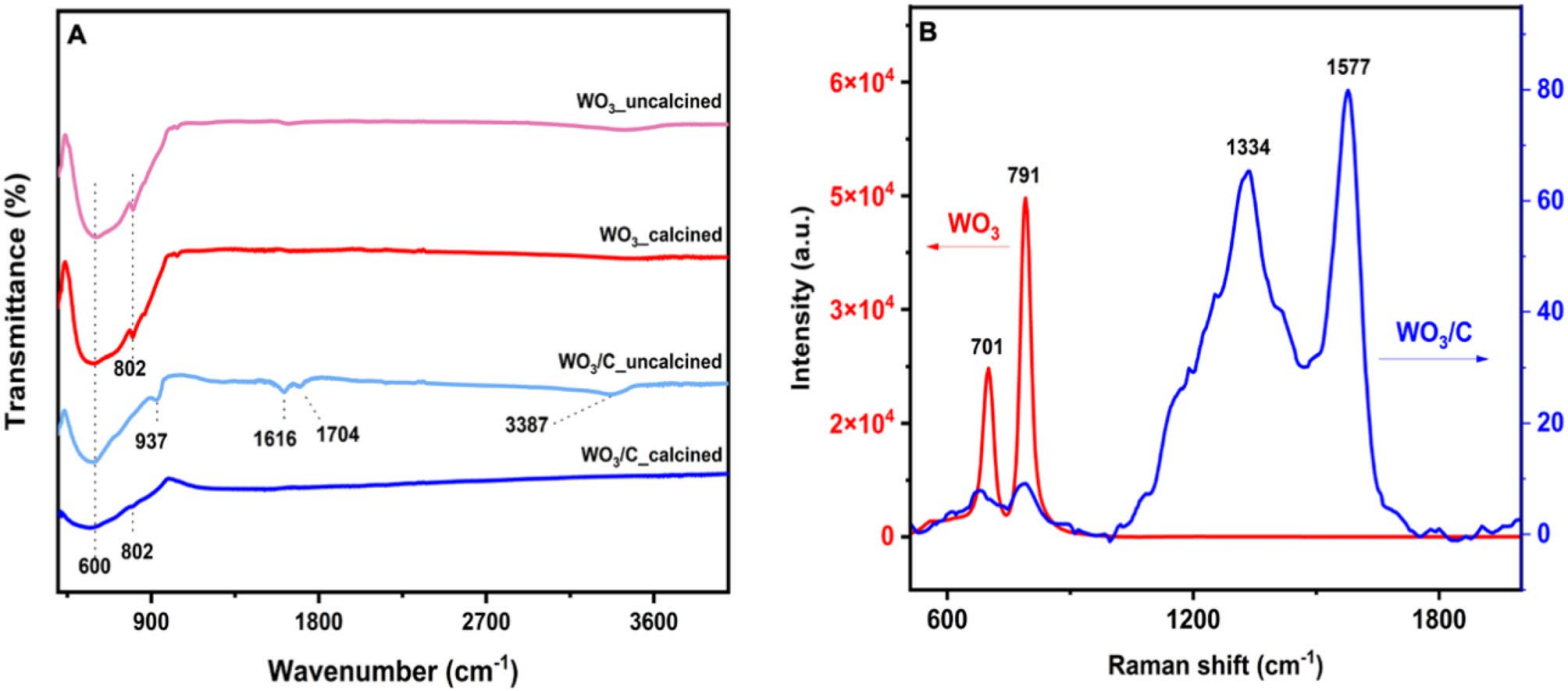
(**A**) FTIR spectra of the samples before and after calcination and (**B**) Raman spectra of calcined WO_3_ and WO_3_/C nanocomposite after calcination.

The FTIR spectra (Fig. 2A) elucidated the different functional groups existing on the surface of the samples. For the uncalcined WO_3_/C, the observed peak at 3387 cm^−1^ corresponded to the stretching vibrations of O–H from water molecules in WO_3_·H_2_O.

This peak was not as prominent in the uncalcined WO_3_ sample, suggesting a significant elimination of crystalline water during the hydrothermal treatment, thereby corroborating with the XRD result. The 1616 cm^−1^ and 1704 cm^−1^ peaks confirmed carbon's presence in the nanocomposite. These spectral bands were associated with the vibrations of C=C and C=O, respectively, and supported the idea that glucose likely underwent aromatization during the hydrothermal treatment^22^. The peaks in the spectral range 500–1000 cm^−1^ were characteristic absorptions of tungsten oxide. The strong peak at about 600 cm^−1^ corresponded to the stretching vibrations of O–W–O. The stretching vibrations of W=O appeared as a sharp shoulder absorption peak at 802 cm^−1^ for the uncalcined and calcined WO_3_ and for the calcined WO_3_/C composite as well, although less sharp. Only the uncalcined WO_3_/C showed a major characteristic band of the terminal oxygen atom (W=O) of the WO_3_⋅H_2_O structure appearing at 937 cm^−1^, again showing agreement with the XRD data^25,27,37^.

The Raman scattering spectra of the calcined samples were also recorded. The spectrum for the control WO_3_ sample exhibited two intense peaks at 701 cm^−1^ and 791 cm^−1^, corresponding to the stretching vibration of tungsten atoms with neighboring oxygen atoms (O–W–O) as shown in (Fig. 2B). These peaks became less intense in the presence of carbon in the WO_3_/C nanocomposite. The prominent peaks at 1334 cm^−1^ (D band) and 1577 cm^−1^ (G band) could be ascribed to the absorption of sp^3^-hybridized carbon and sp^2^-hybridized carbon, respectively. The D band is linked to structural disorder and defects, while the G band indicates the graphitization of carbon. Even though the XRD peaks for graphitic carbon ((002) at 24° and (100) at 43°) were overshadowed by the highly crystalline WO_3_, the Raman spectrum for WO_3_/C confirmed its presence. The intensity ratio of the D to the G peak (I_D_/I_G_) was measured at 0.818, attributing the higher G band to the graphitic clusters in the amorphous composite^3,25,38,39^.

**Figure 3:**
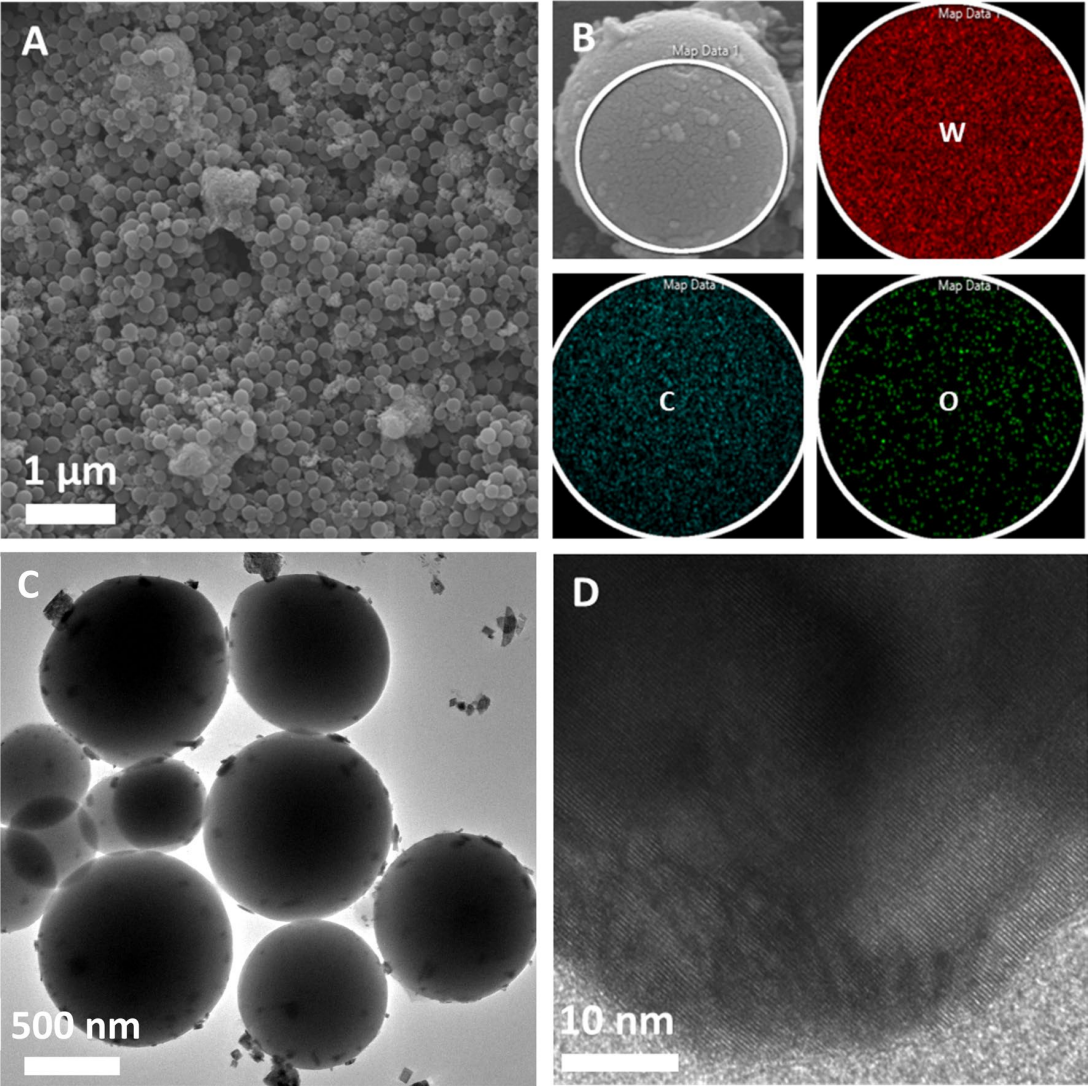
(**A**) Scanning electron microscopy (SEM) images of WO_3_/C. (**B**) Elemental mapping of WO_3_/C (red = W, teal = C; green = O) by energy-dispersive x-ray (EDX) spectroscopy. (**C**) TEM image WO_3_/C and (D) HRTEM of WO_3_ nanocrystal attached on carbon surface.

The nanocomposite morphology was observed by SEM (Fig. 3A,B) and TEM (Fig. 3C,D). Carbon spheres were derived from glucose during the hydrothermal treatment at 180 °C which is higher than the typical glycosylation temperature, resulting in aromatization and carbonization. Glucose molecules underwent dehydration and formed oligosaccharides, resembling a polymerization process. The new carbon–carbon bonds eventually formed the carbon microspheres of > 1.0 µm in diameter. It could be presumed that within the 20 h hydrothermal reaction, the solution reached a critical supersaturation, and a burst of nucleation ensued, crosslinking the previously formed oligosaccharides. This aggregation of glucose consequently acted as a spherical nucleus onto which WO_3_ nanocrystals grew via heterogeneous nucleation^14,22^. This process successfully formed a nanocomposite consisting of tungsten oxide and carbon (WO_3_/C), as confirmed by the elemental mapping of a single sphere by energy-dispersive x-ray (EDX) spectroscopy (Fig. 3B). The EDX mapping spectrum revealed 56.9% C, 37.2% W, and 5.9% O (Supporting Information Fig. S1). The SEM images of WO_3_ synthesized without using glucose are shown in Fig. S2. Additionally, the lack of peaks in the range 1000 cm^−1^ to 1300 cm^−1^ in the FTIR spectrum of the uncalcined WO_3_/C further supported the loading of WO_3_ nanocrystals onto the carbon microspheres. Peaks in this range would have indicated C–OH stretching and OH bending vibrations from residual hydroxy groups. The lack thereof suggested that the hydroxyl groups of glucose have formed hydrogen bonds with WO_3_·H_2_O, which preserved the orthorhombic structure of the latter, as previously discussed in the XRD findings.

**Figure 4:**
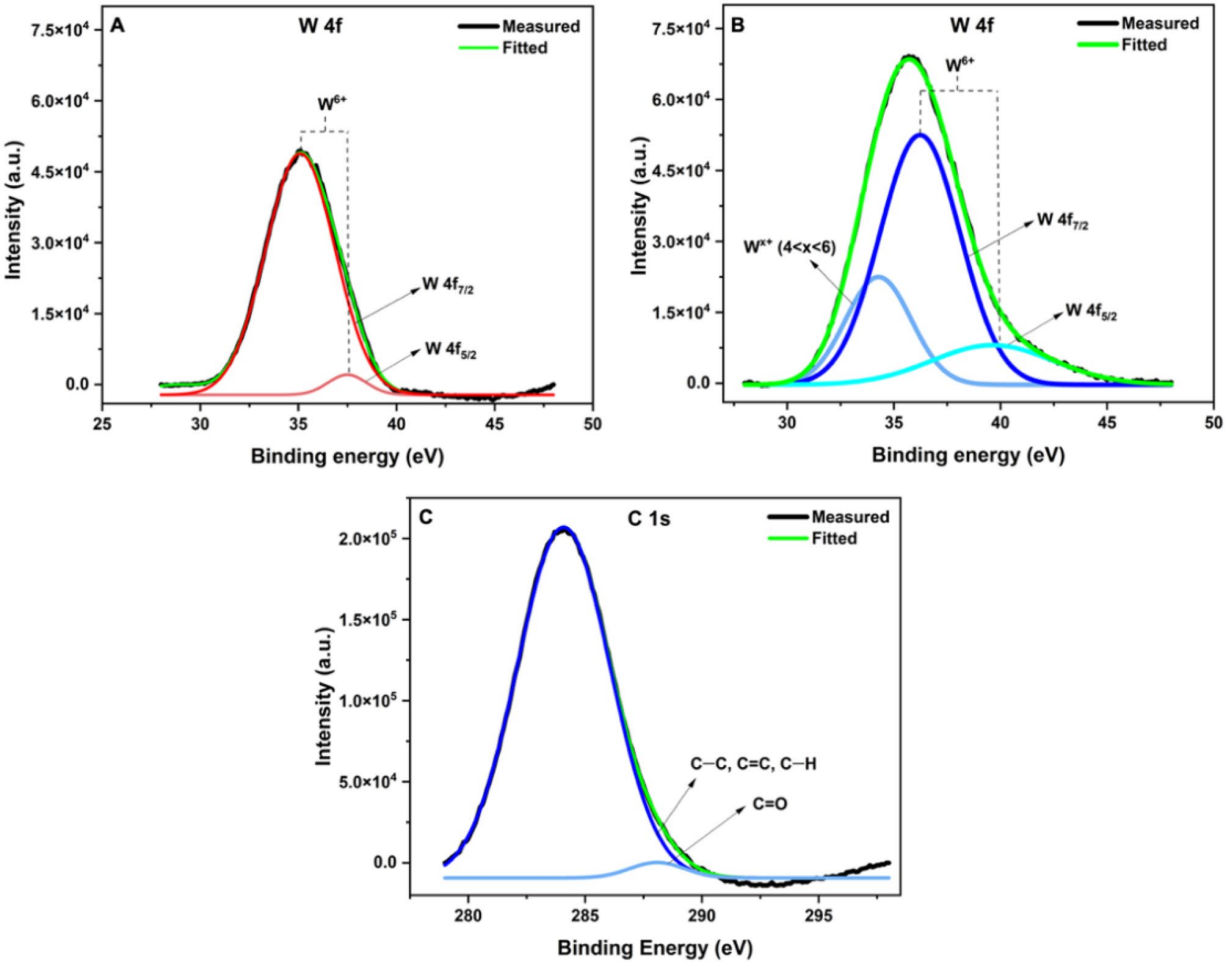
XPS spectra of W 4f of (**A**) WO_3_ and (B) WO_3_/C. (**C**) C 1s spectrum of WO_3_/C nanocomposite.

Results from the elemental mapping by EDX of the WO_3_/C nanocomposites were further confirmed by x-ray photoelectron spectroscopy (XPS). The XPS survey spectrum of the nanocomposite showed the presence of W, O, and C elements (Fig. S3). Furthermore, a comparison of the W 4f. spectra of the WO_3_/C nanocomposite (Fig. 4A) with that of WO_3_ (Fig. 4B) shows that introducing carbon in the nanocomposite altered the chemical state of tungsten. The WO_3_/C nanocomposite exhibited three resolved peaks, whereas WO_3_ alone displayed just a pair of peaks in the deconvoluted spectra. In the WO_3_ spectrum, the peaks at 35.1 eV (W 4f_7/2_) and 37.5 eV (W 4f_5/2_) corresponded to the W^6+^ oxidation state^34,40,41^. These shifted to slightly higher binding energies in WO_3_/C (36.2 eV and 39.6 eV), indicating a change in the chemical environment of tungsten. The nanocomposite also displayed peak broadening, particularly in W 4f_5/2_ (FWHM of WO_3_ = 2.3 eV; WO_3_/C = 6.7 eV), further confirming alterations in the number of chemical bonds^42^. A third peak at 34.3 eV was also present in the nanocomposite and could be ascribed to W^x+^ (where 4 < x < 6). A similar peak was also observed in another work wherein WO_3_-carbon nanotubes showed tetragonal WO_3_ in its XRD^43^, similar to this work. They found that the existence of W^x+^ was beneficial for increasing conductivity and, thereby, electrochemical performance. It is also worth mentioning that the WO_3_/C nanocomposite had higher-intensity W 4f. peaks than the pristine WO_3_. This might be explained by the increase in the effective surface area of the nanocomposite since carbon materials often have higher surface areas than metal oxides. The more intense XPS signals could be due to the larger fraction of the surface being probed during the analysis. This higher intensity was also observed in the O 1s spectra of the samples (Fig. S4). Moreover, the WO_3_/C nanocomposite showed a high-intensity C 1s peak, which deconvoluted to two peaks at 284.1 eV and 288.1 eV (Fig. 4C). The former could be ascribed to C–C, C=C, and C–H bonds while the latter to C=O bonds^44^. The presence of these peaks agrees with the FTIR results and further proves the aromatization of carbon during synthesis.

**Figure 5:**
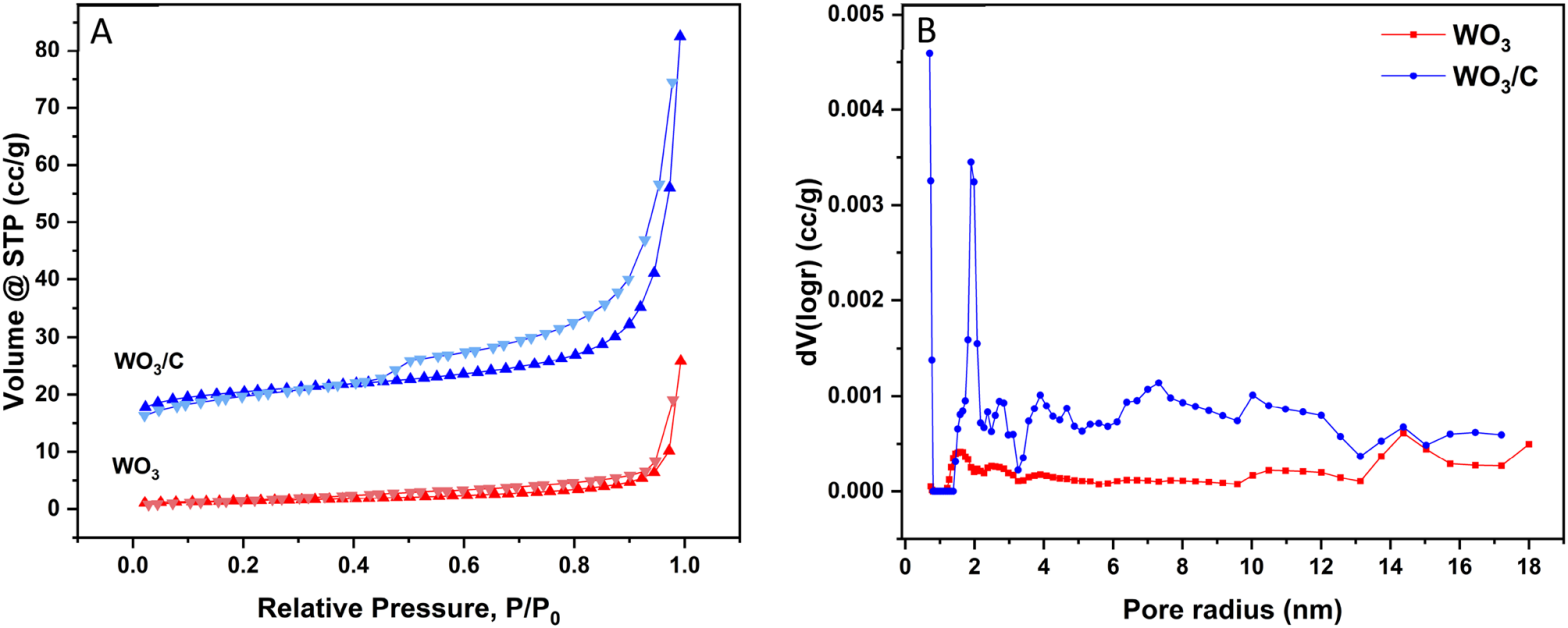
Nitrogen adsorption–desorption isotherms (**A**) and DFT pore size distribution curves (**B**) of WO_3_ and WO_3_/C nanocomposite.

The surface area and pore characteristics were examined by nitrogen adsorption–desorption analysis, as presented in Fig. 5. The isotherm of pure WO_3_ closely resembles a type II isotherm, suggesting that it is an aggregation with predominantly macroporous features^45^. The adsorption amount of N_2_ for the WO_3_/C nanocomposite significantly increased, displaying a type IV isotherm with an apparent hysteresis loop at a relative pressure range of 0.45–0.95, suggesting the presence of abundant mesopores. The presence of such mesopores was further confirmed in the pore size distribution plot, revealing a range of pore radii between 1.5 and 15.0 nm with the highest peak occurring at 2.0 nm. Mesopores (ranging from 2 to 50 nm based on the IUPAC categorization) play a crucial role in enabling the migration of ions towards smaller micropores (those less than 2 nm in size). These facilitate the smooth transportation of ionic substances and the interconnected pore structure supports the formation of an electric double-layer during the charging process ^46^. The surface area and pore volume, calculated by the density functional theory (DFT) method, were 4.1 m^2^/g and 0.02 cc/g for WO_3_ while 58.5 m^2^/g and 0.09 cc/g for WO_3_/C nanocomposite.

**Figure 6:**
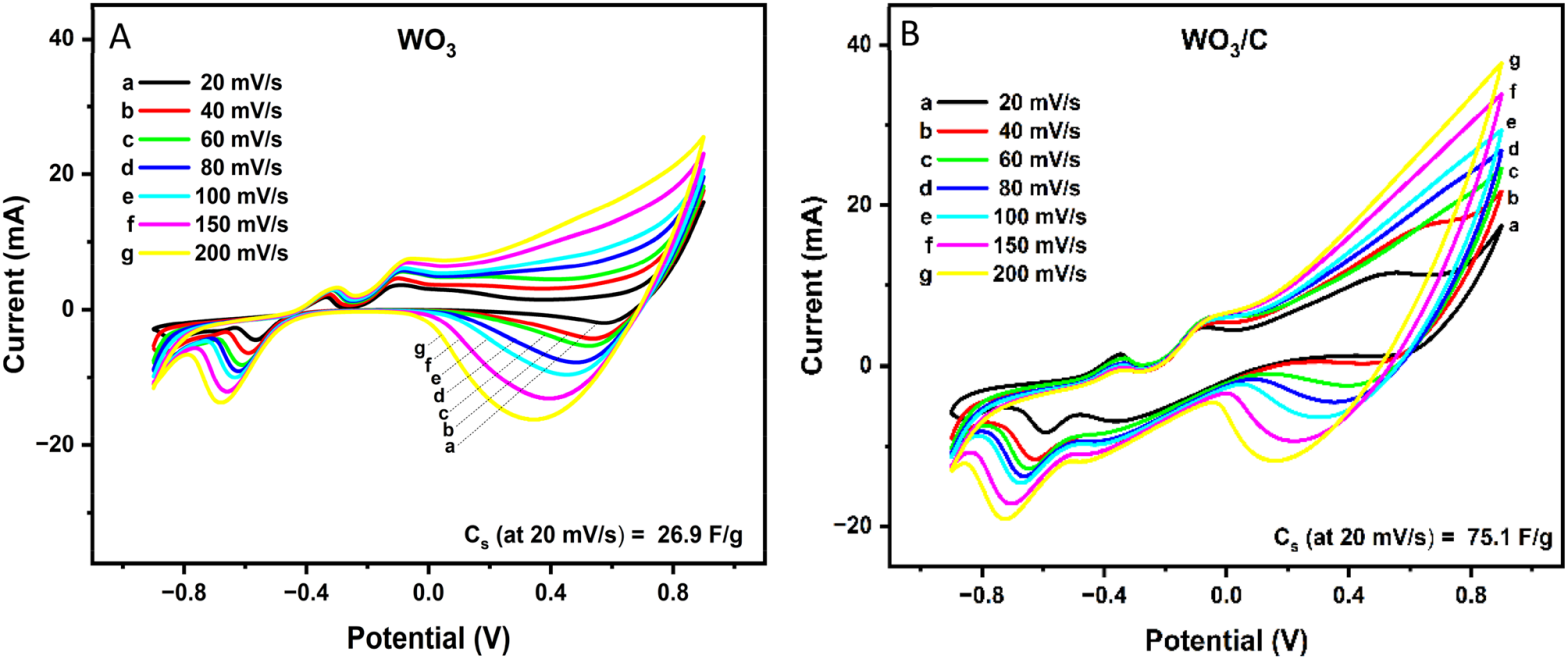
Cyclic voltammetry (CV) curves of (**A**) WO_3_ and (**B**) WO_3_/C nanocomposite at different scan rates.

**Figure 7:**
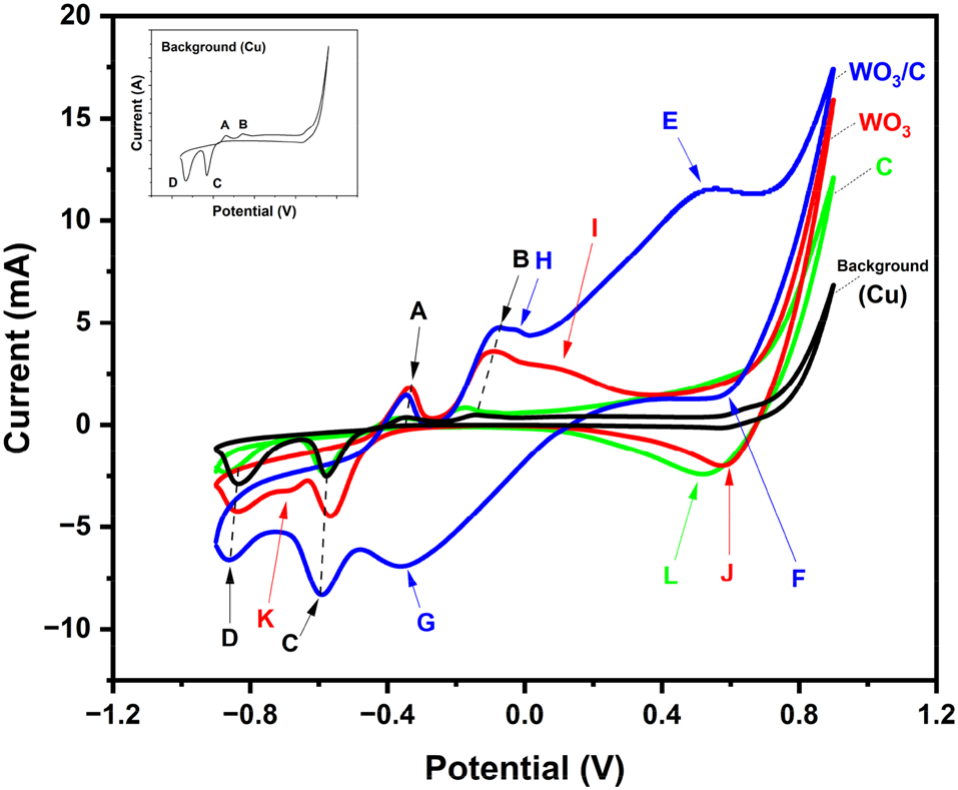
Comparative CV curves at 20 mV/s scan rate of WO_3_/C, WO_3_, C, and background signal from the Cu substrate.

The electrochemical charge storage properties of WO_3_ and WO_3_/C nanocomposite were evaluated using cyclic voltammetry (CV), galvanostatic charge–discharge (GCD) technique, and impedance spectroscopy. Figure 6A and B display the CV curves of the WO_3_ and WO_3_/C electrodes between the potential range of − 0.90 to 0.90 V at different scan rates ranging from 20 mV/s to 200 mV/s. The anodic and cathodic currents increased with higher scan rates, which is a normal occurrence in CV. The comparative CV curves (Fig. 7) at a lower scan rate of 20 mV/s show that the WO_3_/C electrode maintains a larger area under the CV curve compared to WO_3_, indicating better capacitance. Additionally, a control sample containing only carbon particles was also prepared and similarly showed lower capacitance than the nanocomposite (Fig. 7, S2B, S6, S7). Moreover, the quasi-rectangular shape of the CV curve suggests pseudocapacitance. Equation (4) represents the electrochemical charge storage mechanism of WO_3_ in the KOH electrolyte^47^:

4 $${\text{WO}}_{{3}} + x{\text{K}}^{ + } + x{\text{e}}^{-} \leftrightarrow {\text{K}}x{\text{WO}}_{{3}}$$

With background correction from the copper substrate and the carbon control taken into consideration (Fig. 7), the WO_3_/C electrode showed an oxidation peak at 0.56 V to 0.76 V (peak E) and a reduction peak at − 0.20 V to − 0.50 V (peak G). While for the WO_3_ electrode, these peaks appeared at 0.0 V to 0.20 V (peak I) and − 0.60 V to − 0.75 V (peak K). This demonstrated the existence of reversible Faradaic reactions, suggesting an ion intercalation into the crystal structure of the metal oxide^37^. Peaks F and J could not be considered cathodic peaks since this was also present in the carbon control (peak L).

Interestingly, the WO_3_/C electrode showed an extra oxidation peak at 0.0 V to − 0.05 V (peak H), suggesting an additional irreversible Faradaic reaction. The redox behavior of WO_3_ and WO_3_/C aligned with the W 4f. XPS results, revealing two oxidation states for WO_3_/C and only one for WO_3_. The two oxidation peaks of the nanocomposite could be assigned to the electroactivity of W^6+^ and W^x+^ (4 < x < 6). It was also observed that the redox peaks became less pronounced at higher scan rates (Fig. 6), which is due to the rapid charge kinetics caused by the high electric field^37^.

Using Eq. (1), the specific capacitance of the WO_3_ and WO_3_/C electrodes was calculated from the CV data. As shown in Fig. S7, the specific capacitance exponentially increased with decreasing scan rate. At a high scan rate of 200 mV/s, there was only a small difference between the specific capacitance of WO_3_ and WO_3_/C electrodes (14.4 F/g and 16.2 F/g). However, as the scan rate decreased, the difference became more apparent. At 20 mV/s, the specific capacitance of WO_3_/C increased to 75.1 F/g while WO_3_ increased to only 26.8 F/g. The values are comparable to that of reported in the literature (Supporting Information Table 1). The observed increase in capacitance at lower scan rates aligns with the typical rate performance in energy storage devices. Lower scan rates allow better diffusion of electrolyte ions to reach the cavities within the electrode material's internal structure while at higher scan rates, ions may get only surface immersion^47,48^. Overall, the higher capacitance of the WO_3_/C nanocomposite affirmed that it has more pathways for charge diffusion within its structure. These pathways were most likely a synergistic effect of the complex mixed-phase (tetragonal/monoclinic) WO_3_ forming a hierarchical structure with the mesoporous carbon microspheres, resulting in an expanded surface area. Both the presence of mesopores, which decrease ion transport resistance, and the Faradaic reactions significantly enhance electrochemical performance throughout the charging and discharging process^49^.

**Figure 8:**
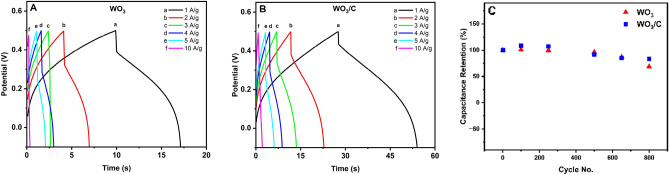
Galvanostatic charge–discharge (GCD) curves for (**A**) WO_3_ and (**B**) WO_3_/C electrodes. (**C**) GCD cycling stability.

Figure 8 shows the GCD curves of the electrodes and their cycling stability. The deviation from the typical triangular shape further supported the pseudocapacitive behavior of the electrode materials. Using Eq. (2), WO_3_/C showed a higher coulombic efficiency than WO_3_. The former exhibited 98.2% efficiency at a current density of 1 A/g while the latter showed 75.8% at the same current density. The nanocomposite material also showed higher energy efficiency (92.8% at 1 A/g) than the pure WO_3_ (65.1%). Additionally, the cycling stability test demonstrated that the WO_3_ and WO_3_/C electrodes retained 68.5% and 83.2% of their capacitance, respectively, after 800 GCD cycles. This enhanced capacity retention in the nanocomposite affirmed that the inclusion of carbon contributes to the material's structural stability. Notably, the particle morphology of WO_3_/C remained unchanged after cycling, as depicted in Figure S8. However, the decrease in the performance for both electrodes could be explained by the possible distortion of the crystal lattice of tungsten oxide which could have adversely affected the charge transport ^40^.

**Figure 9:**
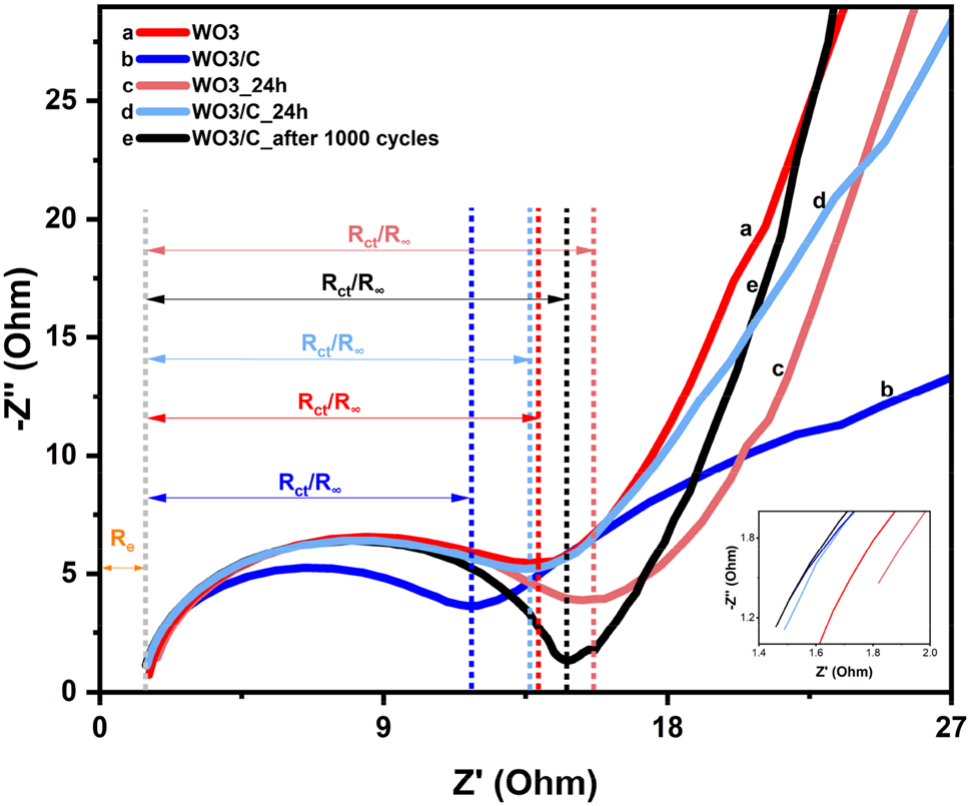
Nyquist plot of the electrochemical impedance. Inset: Emphasis on the difference.

The charge transfer ability and interface resistance of the WO_3_ and WO_3_/C electrodes were studied by electrochemical impedance spectroscopy (EIS). Figure 9 shows the Nyquist plot of EIS measurements performed from 1 Hz to 100 kHz. The semicircular arc in the high-frequency region is indicative of the charge-transfer resistance (R_ct_) attributed to Faradaic reactions at the electrode/electrolyte interface^6,50^. WO_3_/C showed a lower resistance at R_ct_ = 11.7Ω compared to WO_3_ at R_ct_ = 14.0Ω. The arc could also be attributed to bulk electrolyte resistance (R_∞_) while the distance from the imaginary impedance axis (− Z″), to the electrode resistance (R_e_). The sum of these two accounts for the total internal resistance of the electrode^51^. The inset in Fig. 9 shows that WO_3_/C also demonstrated a lower R_e_ than WO_3_. The conductivity was found to be 0.55 S/m for WO_3_ and 0.68 S/m for WO_3_/C. A sloped line related to Warburg resistance or diffuse layer resistance was present at the low-frequency region. From a physical interpretation of this line, steep slopes indicate that the dominating process is electric double-layer (EDL) formation, while it is ion diffusion at low slopes^51^. Interestingly, the WO_3_/C electrode showed a low slope (b in blue), but after 24 h of stabilization in the electrolyte the slope became steeper (d in light blue). But for WO_3_, the slope was already steep (a in red) and did not change much after 24 h (c in light red). This observation supports more channels in the nanocomposite arising from both the inner crystal structure and the amorphous carbon network. The relatively deep insertion of ions leads to a relatively longer time for the EDL to form, and thus ion diffusion was still the dominating process at the beginning^40^. After 24 h, the R_ct_ for WO_3_/C and WO_3_ were 13.6Ω and 15.7Ω, respectively. Moreover, for WO_3_/C, the EIS spectra after 1000 CV cycles showed an R_ct_ of 14.9Ω and the steepest slope, suggesting effective ion physisorption at the electrode/electrolyte interface.

## Conclusion

A facile hydrothermal method successfully prepared a hierarchical nano/microstructure WO_3_/C nanocomposite. Glucose, used as the carbon-source precursor, also influenced the crystal phase transformation of WO_3_. It contained a phase junction of tetragonal/monoclinic WO_3_ uniformly embedded on carbon microspheres and exhibited more oxidation states. Owing to this distinctive structure, the WO_3_/C electrode exhibited better electrochemical performance with a specific capacitance of 75.1 F/g compared to pure WO_3_ with 26.8 F/g at a scan rate of 20 mV/s in 0.1 M KOH. A pseudocapacitive behavior was observed, with WO_3_/C showing a high coulombic efficiency at 98.2% at a current density of 1 A/g. Cyclic voltammetry and impedance spectroscopy results suggested that the nanocomposite's energy storage mechanism showed both Faradaic and non-Faradaic capacitance.

### Supplementary Information

Supplementary Information 1.

Supplementary Information 2.

## Data Availability

The data generated or analyzed during this study are available within the article and its supplementary material. Raw data of XRD, FTIR, Raman, SEM, TEM, XPS, electrochemical impedance, cyclic voltammetry, and galvanostatic charge–discharge are provided in the supplementary material (Raw data). All other data is available from the corresponding author upon request.
