# Supplementary figures and images for: Hydrothermal synthesis of hierarchical microstructure tungsten oxide/carbon nanocomposite for supercapacitor application
Source: Sci Rep. 2023 Dec 8;13:21732. doi: 10.1038/s41598-023-48958-w (PMC10709354; doi:10.1038/s41598-023-48958-w)

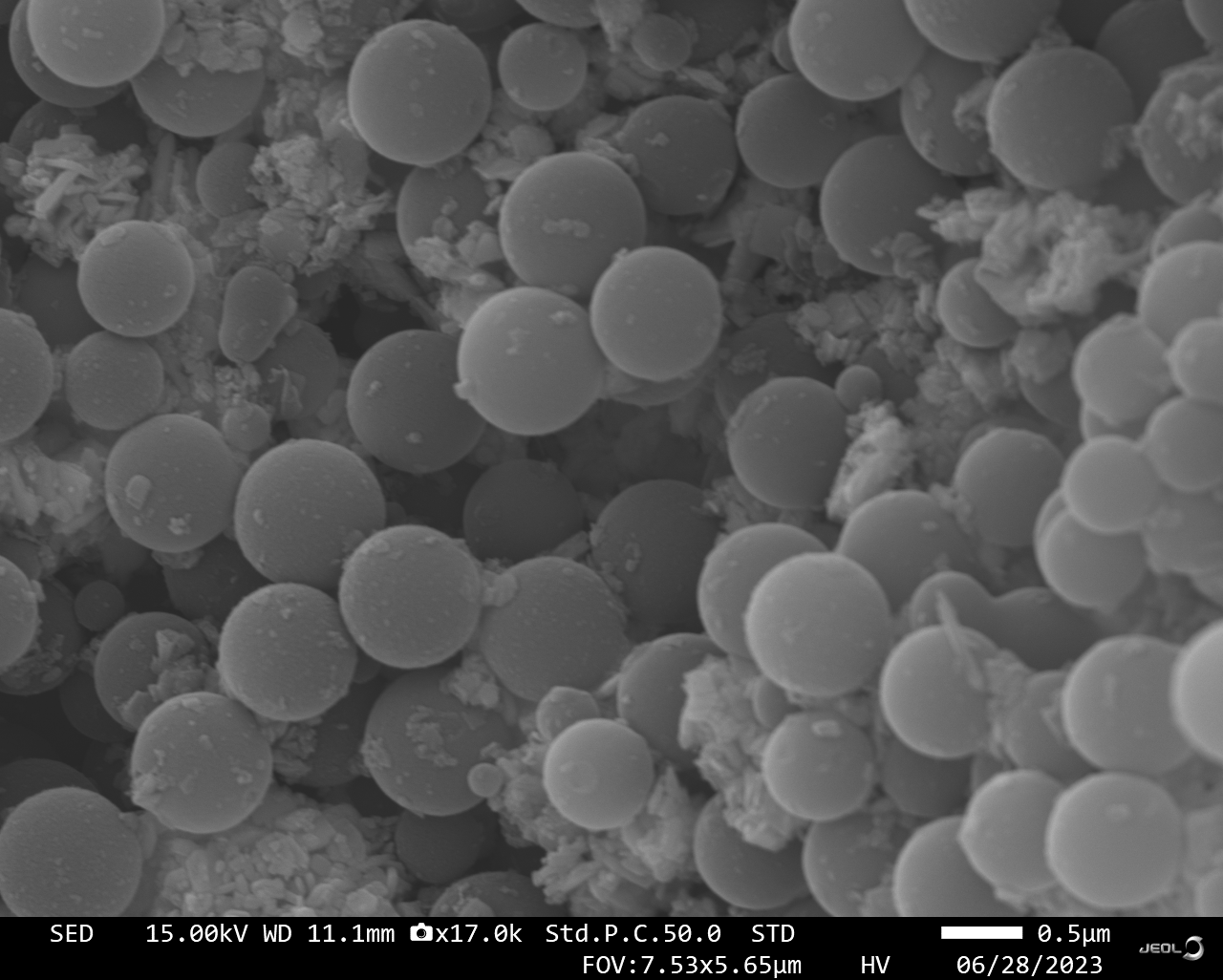

Supplement: Supplementary file 1 — Supplementary Information 1. [file 41598_2023_48958_MOESM1_ESM.zip › Raw Data/4 SEM/WO3-C_1.tif]

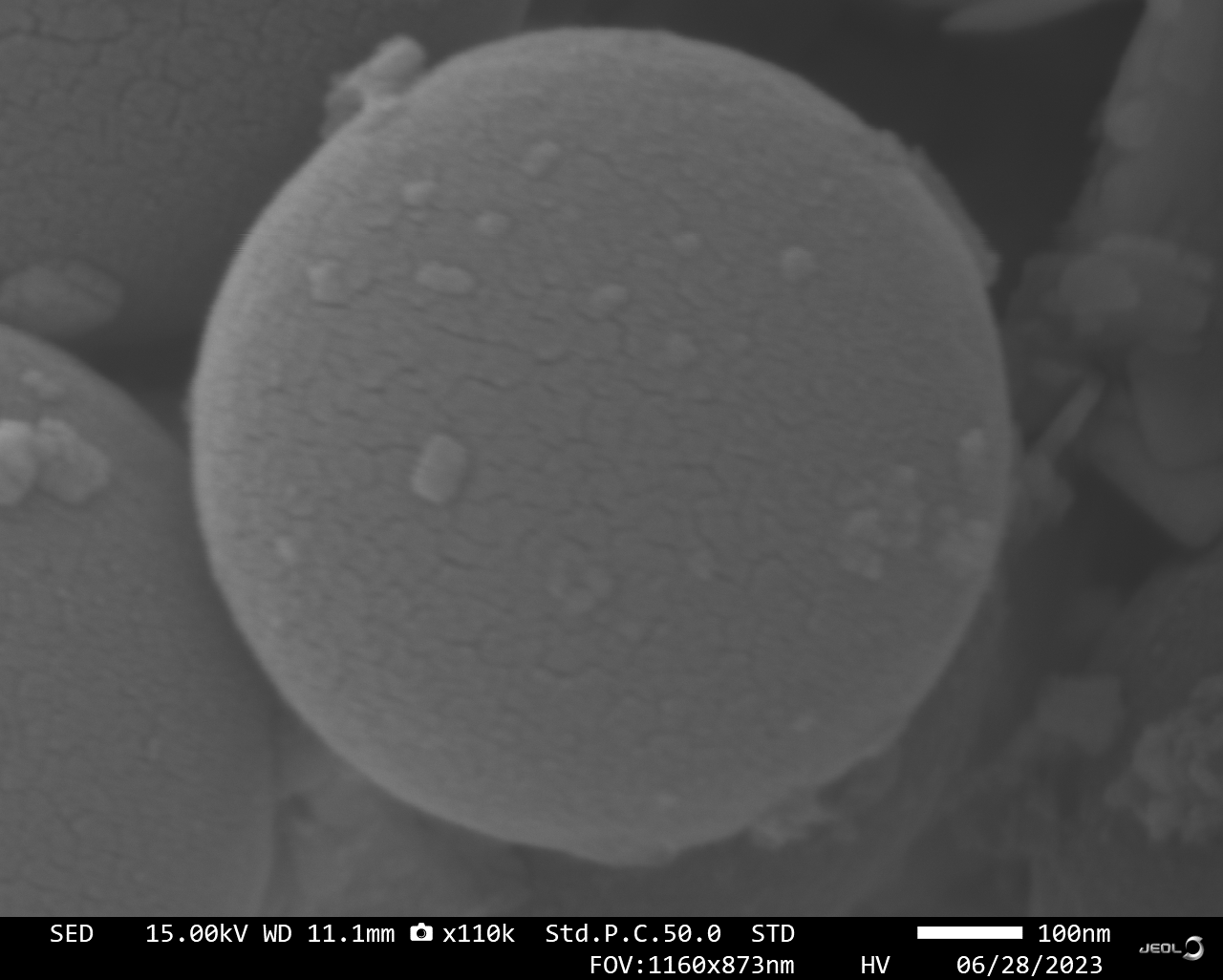

Supplement: Supplementary file 1 — Supplementary Information 1. [file 41598_2023_48958_MOESM1_ESM.zip › Raw Data/4 SEM/WO3-C_2.tif]

# Click here to enter text.


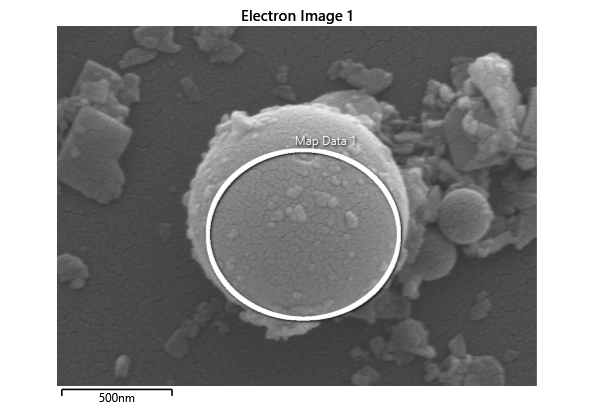


Settings

Phases for Acquisition


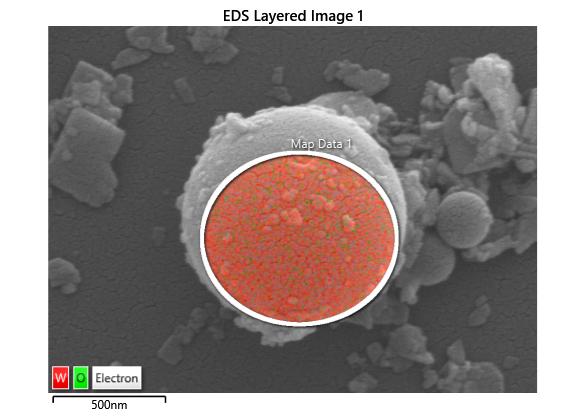


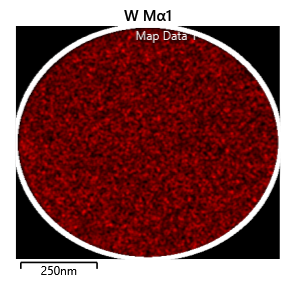


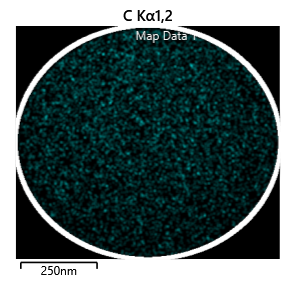


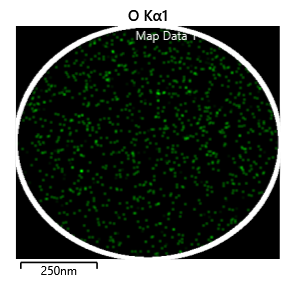

Supplement: Supplementary file 1 — Supplementary Information 1. [file 41598_2023_48958_MOESM1_ESM.zip › Raw Data/4 SEM/WO3-C EDX.docx]

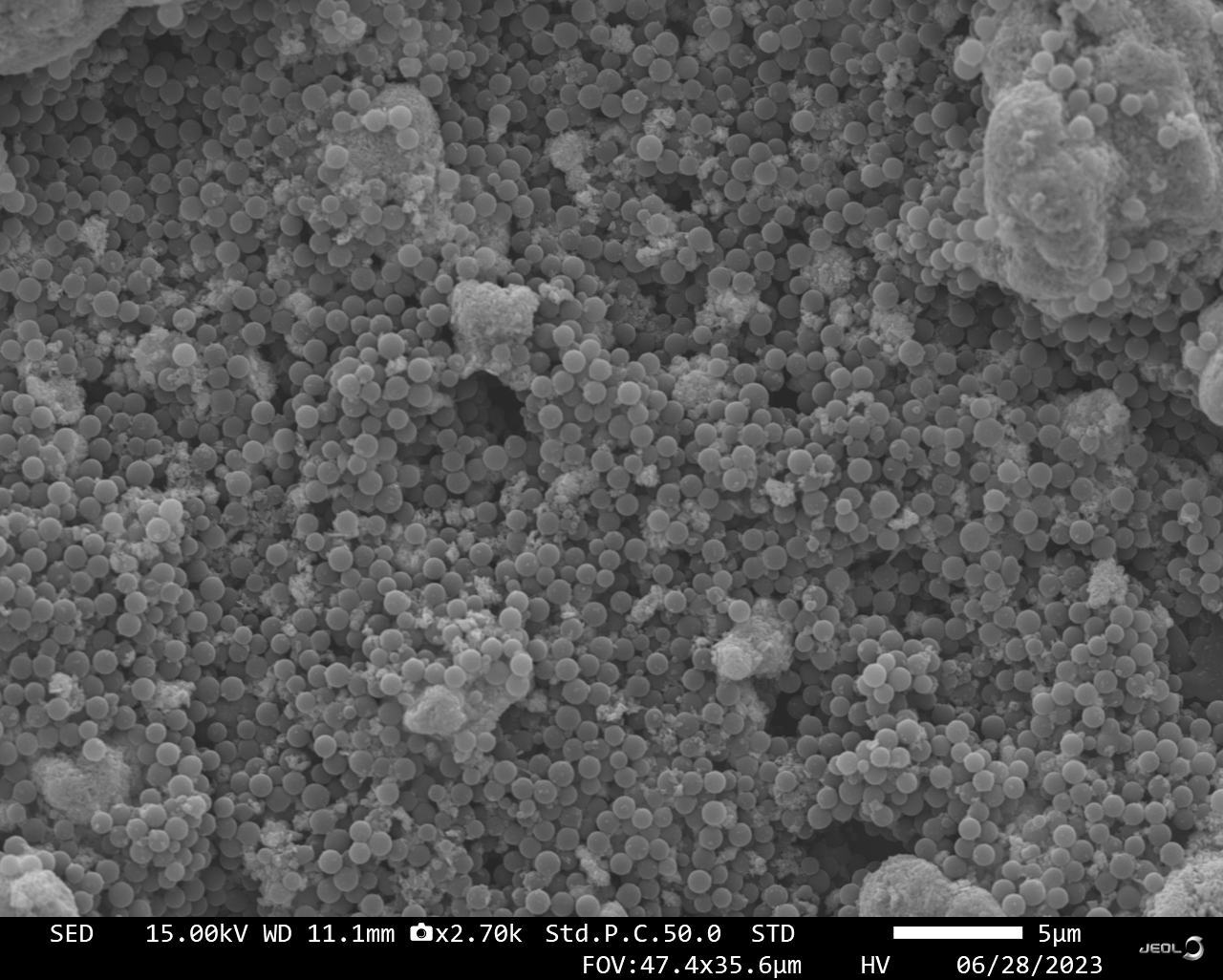

Supplement: Supplementary file 1 — Supplementary Information 1. [file 41598_2023_48958_MOESM1_ESM.zip › Raw Data/4 SEM/WO3-C_3.tif]

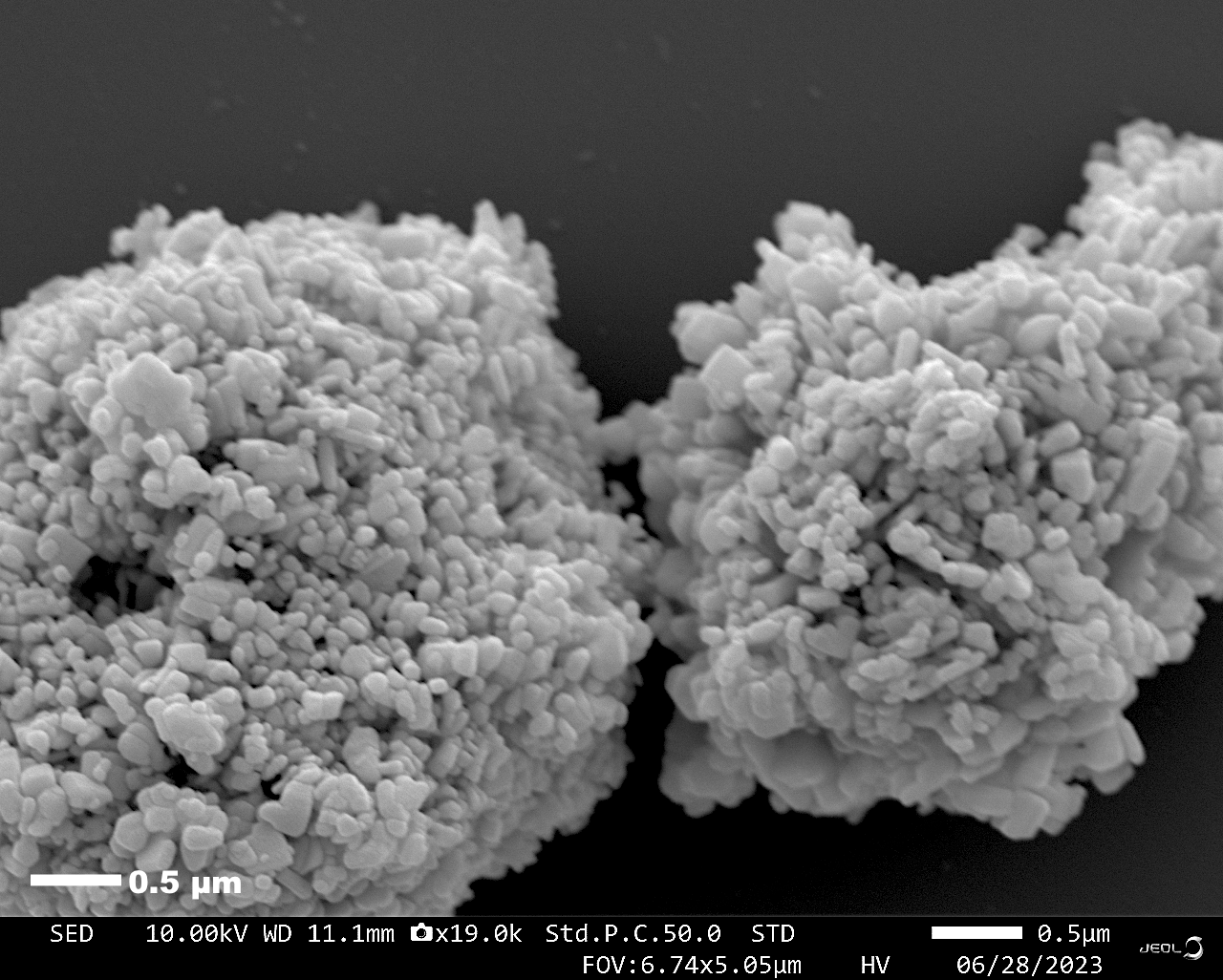

Supplement: Supplementary file 1 — Supplementary Information 1. [file 41598_2023_48958_MOESM1_ESM.zip › Raw Data/4 SEM/WO3.tif]

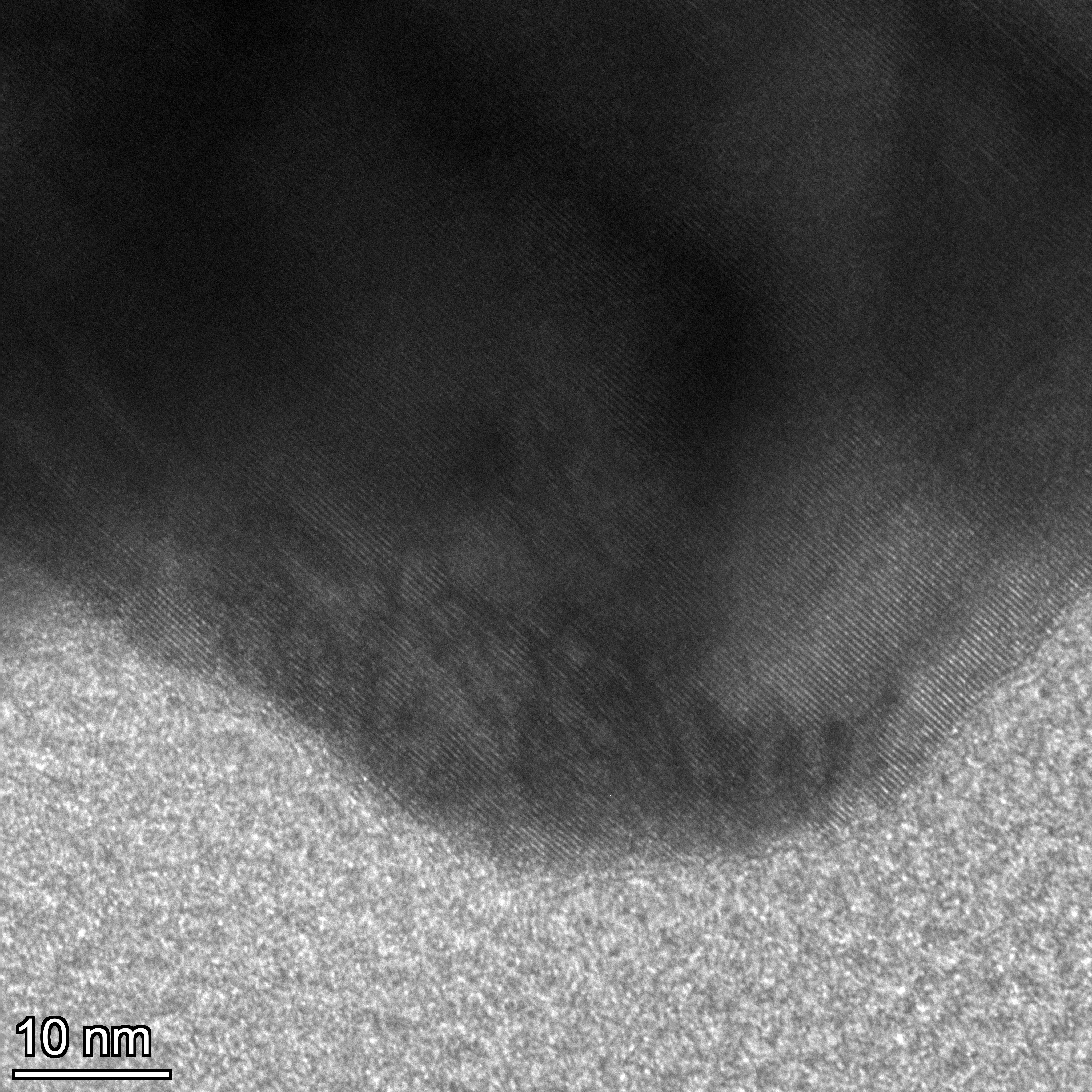

Supplement: Supplementary file 1 — Supplementary Information 1. [file 41598_2023_48958_MOESM1_ESM.zip › Raw Data/4 SEM/WO3-C 1001.jpg]

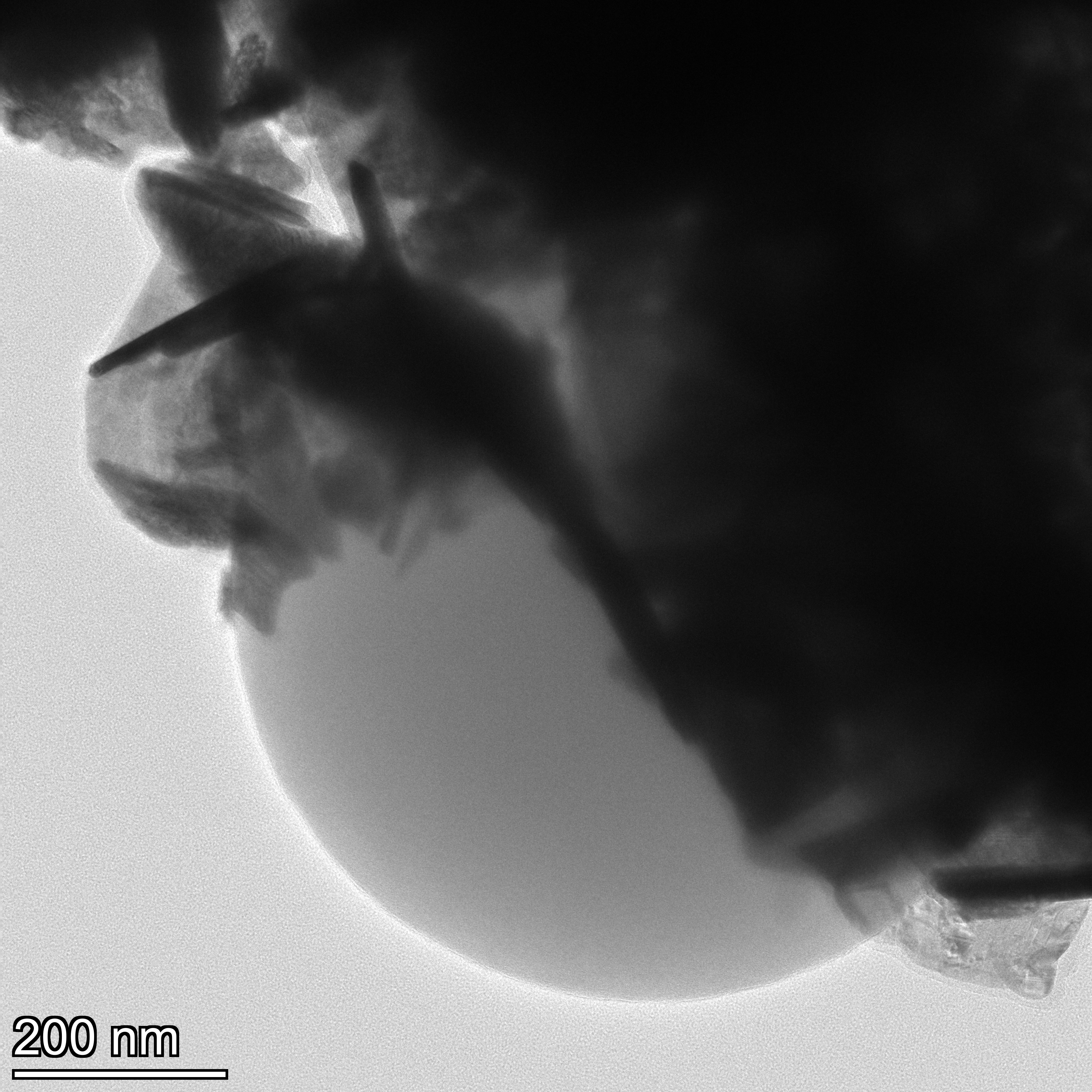

Supplement: Supplementary file 1 — Supplementary Information 1. [file 41598_2023_48958_MOESM1_ESM.zip › Raw Data/4 SEM/WO3-C 1013.jpg]
