# Supplementary material for: Hydrothermal synthesis of hierarchical microstructure tungsten oxide/carbon nanocomposite for supercapacitor application
Source: Sci Rep. 2023 Dec 8;13:21732. doi: 10.1038/s41598-023-48958-w (PMC10709354; doi:10.1038/s41598-023-48958-w)
Supplement: Supplementary file 2 — Supplementary Information 2. [file 41598_2023_48958_MOESM2_ESM.docx]

**Supporting Information**


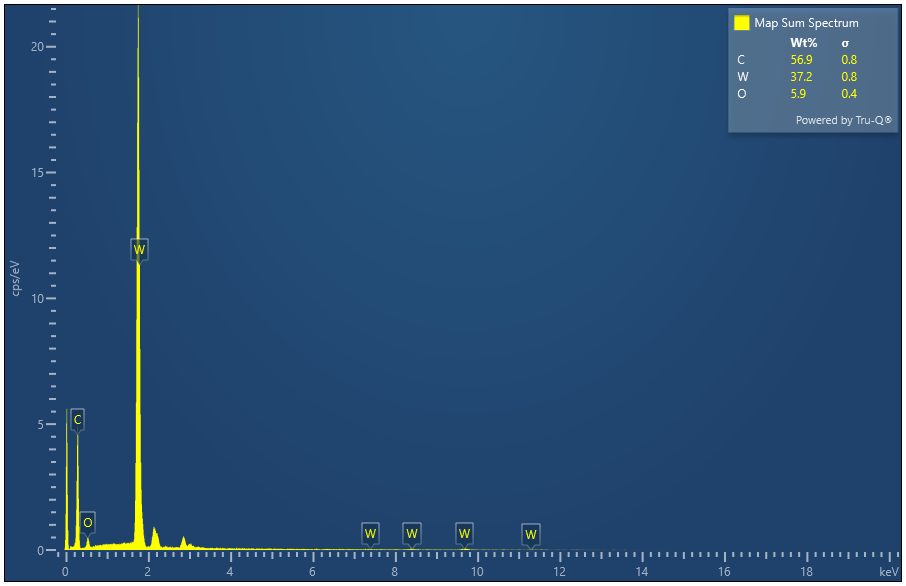


**Fig. S1.** EDX analysis of WO_3_/C nanocomposite.

**Fig. S2.** SEM Image of (A) pure WO_3_ and (B) carbon control samples.


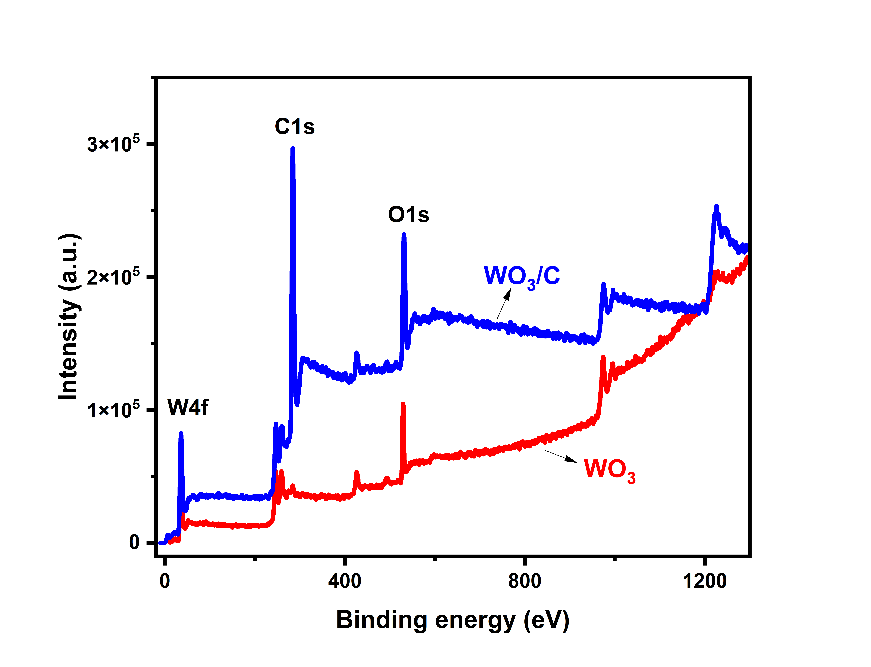


**Fig. S3.** XPS survey spectra of WO_3_ and WO_3_/C nanocomposite.


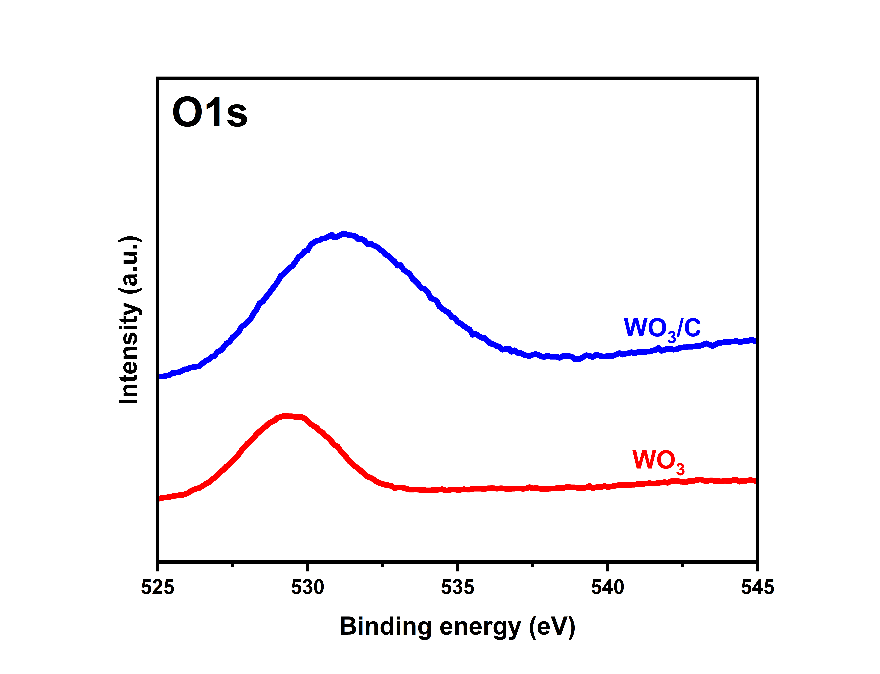


**Fig. S4.** O 1s spectra of WO_3_ and WO_3_/C nanocomposite.


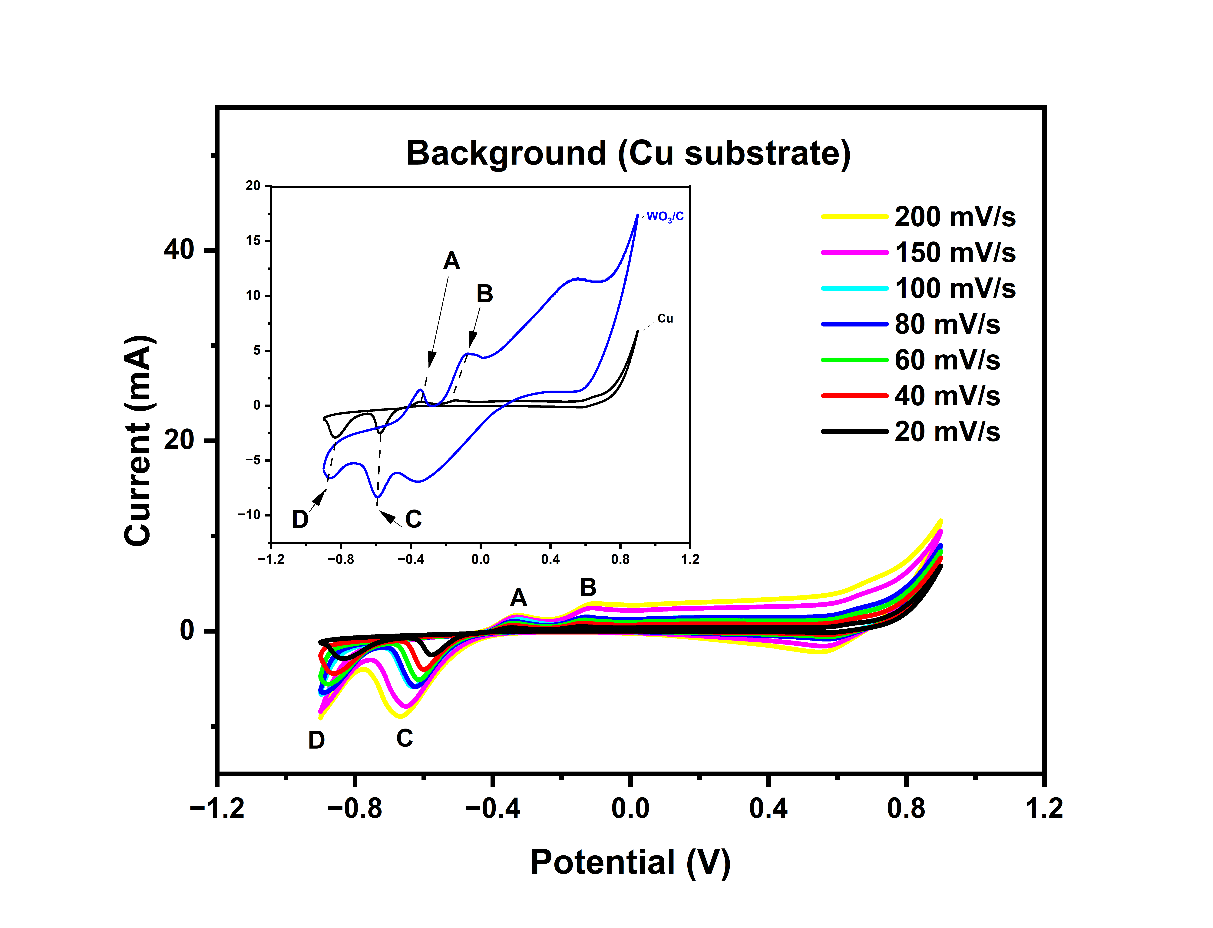


**Fig. S5.** Cyclic voltammetry (CV) curves of the Cu collector at different scan rates. Inset: Comparative CV curves of Cu and the WO_3_/C nanocomposite at 20 mV/s

Although its signal was significantly lesser than the working electrodes, the Cu current collector still exhibited electrochemical activity. Therefore, its response should be taken into account. It showed two pairs of redox peaks which are labeled A, B, C, and D in Fig. S5 (also compared with the CV curve of the WO_3_/C composite in the inset). Peak A is related to the formation of Cu_2_O via a two-electron direct oxidation of copper [1], [2]:

2 Cu + 2 OH^–^  → Cu_2_O + H_2_O + 2e^-^

With a continued increase in potential, the anodic curve showed another notable surge in current (peak B), signifying further oxidation [1]:

Cu_2_O + 2 OH^–^  + H_2_O → 2 Cu(OH)_2_ + 2e^-^

This is followed by a subsequent dehydration step leading to CuO [1], [3]:

Cu(OH)_2_ → CuO + H_2_O

During the reverse scan, moving from the positive potential to the negative potential end, two peaks, identified as peak C and peak D, were once more observed. Peak C primarily results from the reduction of CuO into metallic copper while peak D corresponds to the reduction of Cu(OH)_2_ to Cu_2_O [1]. Cu_2_O largely remains on the surface [1], [4], [5].

The reported specific capacitance values for WO_3_ and WO_3_/C in this work were background-corrected with the contribution from the Cu collector. The capacitance of Cu ranged from 0.48 to 1.09 F/g at scan rates of 20 to 200 mV/s.


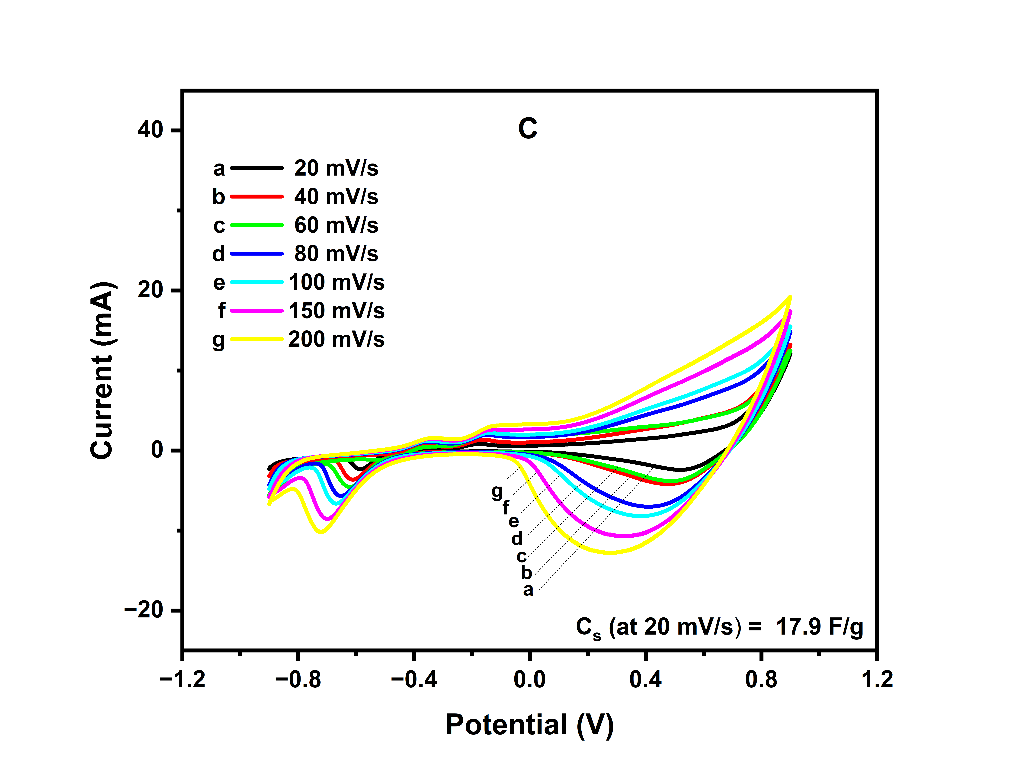


**Fig. S6.** Cyclic voltammetry (CV) curves of carbon control sample at different scan rates.


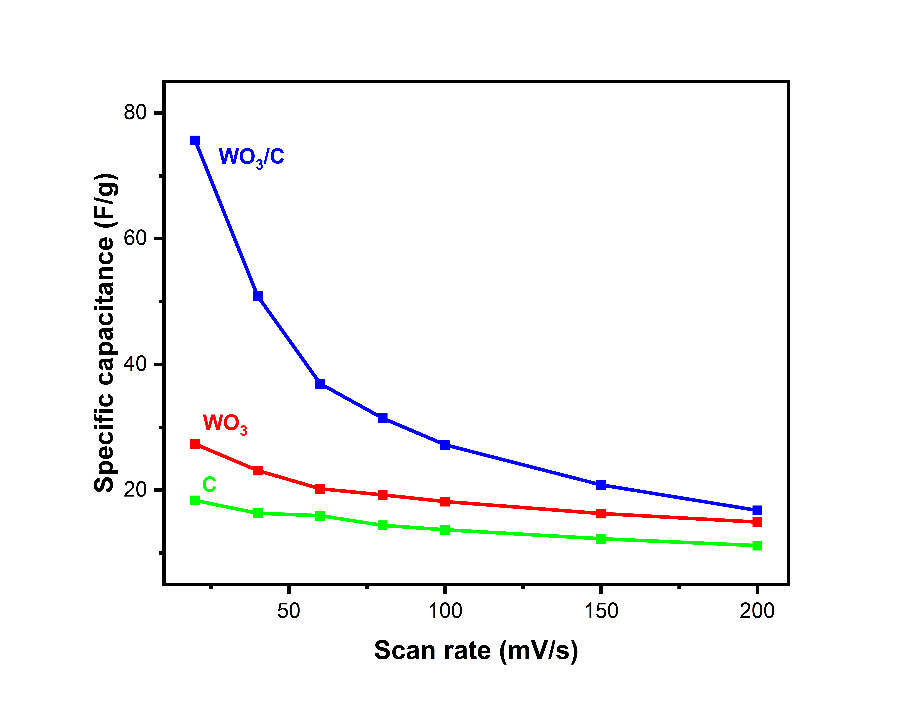


**Fig. S7.** Specific capacitance as a function of scan rate.

**Fig. S8.** Particle morphology after GCD cycling of WO_3_/C nanocomposite.

***Table 1.*** *Performance of carbon-based and WO_3_-based supercapacitors reported in the literature.*

| Material | Method | Potential window  (V) | Scan rate  (mV/s) | Current density  (A/g) | Electrolyte | Specific capacitance | Ref. |
| --- | --- | --- | --- | --- | --- | --- | --- |
| WO_3_/C | Hydrothermal | -0.9 –0.9 | 20 | - | 0.1 M KOH | 75.1 F/g | This work |
| Aligned carbon nanotube array (ACNTA) | Chemical vapor deposition | - | - | 0.05 | EMIBF_4_ | 14.1 F/g | [6] |
| Graphene | Modified Hummers method | 0.0–3.0 | - | - | TEABF_4_ | 35.96 F/g (device) | [7] |
| WO_3_·H_2_O sheets | Wet chemical | - | - | 2.0 A/g | 1 M Na_2_SO_4_ | 71.25 F/g | [8] |
| Co–WO_3_/ fCNT | Solution growth + microwave irradiation | - | - | 1.0 A/g | 2 M KOH | 60.14 F/g | [9] |
| WO_3-x_ /C | Evaporation-induced self-assembly | -0.2–0.8 | 1 | - | 2 M H_2_SO_4_ | 81 F/g | [10] |
| CNT+V_2_O_5_ | Hydrothermal | 0.0–0.8 | 100 | - | 0.1 M KCl | 26.28 F/g  (anodic)  18.96 F/g  (cathodic) | [11] |
| CNT/RuO_2_ | Sol-gel | 0.0–1.0 | 20 | - | 1 M H_2_SO_4_ | 70.0 F/g | [12] |
| Carbon/ siloxene/  Ni | Topochemical method + slurry coating | 0.0–3.0 | - | - | 1 M TEABF_4_ | 24.65 F/g (device) | [13] |
| rGO/ZnO/ rGO sandwich | Chemical vapor deposition | -1.0–0.0 | 10 | - | 1 M KCl | 51.6 F/g | [14] |

**References**

[1] S. D. Giri and A. Sarkar, “Electrochemical Study of Bulk and Monolayer Copper in Alkaline Solution,” *J Electrochem Soc*, vol. 163, no. 3, pp. H252–H259, 2016, doi: 10.1149/2.0071605jes.

[2] M. J. Dignam and D. B. Gibbs’, “Anodic oxidation of copper in alkaline solution,” 1970.

[3] S. M. A. El Haleem and B. G. Ateya, “Cyclic voltammetry of copper in sodium hydroxide solutions,” *J. Electroanal. Chem.*, no. 117, pp. 309–319, 1981.

[4] J. Ambrose, R. G. Barradas, and D. W. Shoesmith, “INVESTIGATIONS OF COPPER IN AQUEOUS ALKALINE SOLUTIONS BY CYCLIC VOLTAMMETRY,” 1973.

[5] G. M. Brisard, J. D. Rudnicki, F. Mclarnon, and E. J. Cairns, “APPLICATION OF PROBE BEAM DEFLECTION TO STUDY THE ELECTROOXIDATION OF COPPER IN ALKALINE MEDIA,” 1995.

[6] H. Zhang, G. Cao, Y. Yang, and Z. Gu, “Comparison Between Electrochemical Properties of Aligned Carbon Nanotube Array and Entangled Carbon Nanotube Electrodes,” *J Electrochem Soc*, vol. 155, no. 2, p. K19, 2008, doi: 10.1149/1.2811864.

[7] S. Manoharan, K. Krishnamoorthy, A. Sathyaseelan, and S. J. Kim, “High-power graphene supercapacitors for the effective storage of regenerative energy during the braking and deceleration process in electric vehicles,” *Mater Chem Front*, vol. 5, no. 16, pp. 6200–6211, Aug. 2021, doi: 10.1039/d1qm00465d.

[8] P. A. Le, V. Q. Le, T. L. Tran, N. T. Nguyen, and T. V. B. Phung, “Computation and Investigation of Two-Dimensional WO3·H2O Nanoflowers for Electrochemical Studies of Energy Conversion and Storage Applications,” *ACS Omega*, vol. 7, no. 12, pp. 10115–10126, Mar. 2022, doi: 10.1021/acsomega.1c06150.

[9] R. Dhilip Kumar, Y. Andou, and S. Karuppuchamy, “Facile synthesis of Co–WO3/functionalized carbon nanotube nanocomposites for supercapacitor applications,” *Journal of Materials Science: Materials in Electronics*, vol. 28, no. 7, pp. 5425–5434, Apr. 2017, doi: 10.1007/s10854-016-6203-9.

[10] C. Jo *et al.*, “Block-copolymer-assisted one-pot synthesis of ordered mesoporous WO 3-x/carbon nanocomposites as high-rate-performance electrodes for pseudocapacitors,” *Adv Funct Mater*, vol. 23, no. 30, pp. 3747–3754, Aug. 2013, doi: 10.1002/adfm.201202682.

[11] M. Jayalakshmi, M. M. Rao, N. Venugopal, and K. B. Kim, “Hydrothermal synthesis of SnO2-V2O5 mixed oxide and electrochemical screening of carbon nano-tubes (CNT), V2O5, V2O5-CNT, and SnO2-V2O5-CNT electrodes for supercapacitor applications,” *J Power Sources*, vol. 166, no. 2, pp. 578–583, Apr. 2007, doi: 10.1016/j.jpowsour.2006.11.025.

[12] J. H. Park, J. M. Ko, and O. Ok Park, “Carbon Nanotube/RuO[sub 2] Nanocomposite Electrodes for Supercapacitors,” *J Electrochem Soc*, vol. 150, no. 7, p. A864, 2003, doi: 10.1149/1.1576222.

[13] K. Krishnamoorthy, M. S. P. Sudhakaran, P. Pazhamalai, V. K. Mariappan, Y. S. Mok, and S. J. Kim, “A highly efficient 2D siloxene coated Ni foam catalyst for methane dry reforming and an effective approach to recycle the spent catalyst for energy storage applications,” *J Mater Chem A Mater*, vol. 7, no. 32, pp. 18950–18958, 2019, doi: 10.1039/c9ta03584b.

[14] G. Guo *et al.*, “Sandwiched nanoarchitecture of reduced graphene oxide/ZnO nanorods/reduced graphene oxide on flexible PET substrate for supercapacitor,” *Appl Phys Lett*, vol. 99, no. 8, Aug. 2011, doi: 10.1063/1.3629789.
